# Reversible lysine acetylation is involved in DNA replication initiation by regulating activities of initiator DnaA in *Escherichia coli*

**DOI:** 10.1038/srep30837

**Published:** 2016-08-03

**Authors:** Qiufen Zhang, Aiping Zhou, Shuxian Li, Jinjing Ni, Jing Tao, Jie Lu, Baoshan Wan, Shuai Li, Jian Zhang, Shimin Zhao, Guo-Ping Zhao, Feng Shao, Yu-Feng Yao

**Affiliations:** 1 Laboratory of Bacterial Pathogenesis, Institutes of Medical Sciences, Shanghai Jiao Tong University School of Medicine, Shanghai 200025, China; 2 Department of Laboratory Medicine, Shanghai East Hospital, Tongji University School of Medicine, Shanghai 200120, China; 3 Department of Infectious Diseases, Ruijin Hospital, Shanghai Jiao Tong University School of Medicine, Shanghai 200025, China; 4 Department of Pathophysiology, Key Laboratory of Cell Differentiation and Apoptosis of Chinese Ministry of Education, Shanghai Jiao Tong University, School of Medicine, Shanghai 200025, China; 5 State Key Lab of Genetic Engineering & Institutes of Biomedical Sciences, Department of Microbiology and Microbial Engineering, School of Life Sciences, Fudan University, Shanghai 200438, China; 6 Key Laboratory of Synthetic Biology, Institute of Plant Physiology and Ecology, Shanghai Institutes for Biological Sciences, Chinese Academy of Sciences, Shanghai 200032, China; 7 National Institute of Biological Sciences, Beijing 102206, China

## Abstract

The regulation of chromosomal replication is critical and the activation of DnaA by ATP binding is a key step in replication initiation. However, it remains unclear whether and how the process of ATP-binding to DnaA is regulated. Here, we show that DnaA can be acetylated, and its acetylation level varies with cell growth and correlates with DNA replication initiation frequencies in *E. coli*. Specifically, the conserved K178 in Walker A motif of DnaA can be acetylated and its acetylation level reaches the summit at the stationary phase, which prevents DnaA from binding to ATP or *oriC* and leads to inhibition of DNA replication initiation. The deacetylation process of DnaA is catalyzed by deacetylase CobB. The acetylation process of DnaA is mediated by acetyltransferase YfiQ, and nonenzymatically by acetyl-phosphate. These findings suggest that the reversible acetylation of DnaA ensures cells to respond promptly to environmental changes. Since Walker A motif is universally distributed across organisms, acetylation of Walker A motif may present a novel regulatory mechanism conserved from bacteria to eukaryotes.

DNA replication initiation is an essential step in cell proliferation across all domains of life. In *Escherichia coli*, DNA replication is initiated by DnaA forming initiation complex with *oriC* region, which is analogous to the origin recognition complex (ORC) associated with eukaryotic replication origins^1^,^2^. The *oriC* contains an AT-rich region that facilitates DNA duplex unwinding and a DnaA assembly region bearing high-affinity, moderate-affinity and low-affinity DnaA-binding sites, called DnaA boxes^1^.

DnaA belongs to the AAA + ATPase protein family. It is a modular protein carrying four domains and domain III has ATP-binding and hydrolysis activity, as well as an independent oligomerization activity^3^. DnaA has a high affinity for both ATP and ADP^4^. ATP-DnaA is active for opening the *oriC* duplex whereas ADP-DnaA is not^5^. The cellular ATP-DnaA level fluctuates during the cell cycle and peaks at the time of replication initiation^6^. After initiation, DnaA-bound ATP is hydrolyzed into ADP in a manner dependent on Hda and DNA-loaded β-clamp, a subunit of DNA polymerase III holoenzyme. This replication-coupled negative feedback mechanism is called RIDA (Regulatory Inactivation of DnaA)^7^. A second DnaA hydrolysis system to support RIDA is called DDAH (*datA*-dependent ATP-DnaA hydrolysis)^8^. DDAH occurs at the post-initiation stage when RIDA is activated. Both RIDA and DDAH repress extra initiation and ensure cell cycle-coordinated replication.

In bacteria cells, replication initiation is strictly coupled with the cellular growth rate. Bacterial growth rate is becoming progressively slower during the transition from the logarithmic phase to stationary phase, when cells become smaller and genome equivalents are reduced, the inter-initiation time is prolonged^9^,^10^,^11^. Under this condition, the cell cycle-coordinated RIDA and DDAH fail to decrease ATP-DnaA level^6^. However, the amount of DnaA is relatively stable during the whole cell growth phase^12^. It seems that it is inadequate to restraining the ATP-DnaA level at the stationary phase when RIDA and DDAH are ineffective. Therefore, there might exist other mechanisms restraining the ATP-DnaA level to coordinate the cell growth rate.

Post-translational modification (PTM) of proteins is an evolutionarily conserved strategy for the efficient and economic control of their biological activities, allowing for quick response to environmental changes^13^. One of the most important PTM is the lysine acetylation which affects diverse protein properties, including enzyme activity, DNA-protein interactions, subcellular localization, transcriptional activity, and protein stability^14^,^15^.

With the development of high throughput proteomic techniques, more bacterial proteins including many central metabolism enzymes were found to be acetylated^16^,^17^,^18^,^19^,^20^,^21^. Interestingly, it has been reported that gene transcription was regulated by acetylation, such as α-CTD of RNA polymerase and transcriptional regulator RcsB^22^,^23^. We recently found that a two component system regulator PhoP could be acetylated, and acetylation inhibits PhoP DNA-binding ability and further regulates Salmonella virulence^24^.

The ε-amino group of a lysine residue can be acetylated by GCN5-related N-acetyltransferase YfiQ^25^,^26^. In addition, AcP (acetyl phosphate) can transfer the acetyl group to proteins nonenzymatically and plays a critical role in protein acetylation in *E. coli*^18^,^19^. As a member of the sirtuin family of NAD^+^- dependent deacetylases, CobB is the predominate deacetylase in *E. coli* and can remove acetyl groups at both enzymatic and nonenzymatic lysine acetylation substrate sites^27^,^28^.

Both HstI and Sir2p are histone deacetylases (HDACs) in eukaryotes. It has been found that the deacetylation of H4K5 by HstI is important for full initiation capacity of some origins and Sir2p negatively regulates replication initiation in yeast^29^,^30^,^31^. These results suggest that the protein acetylation participates in the regulation of DNA replication in eukaryotes. However, it remains unclear whether acetylation is involved in DNA replication initiation in bacteria.

Here, we show that DnaA is acetylated in *E. coli* and its acetylation level fluctuates in a growth phase-dependent manner. Lysine 178 (K178) located in Walker A motif is a key acetylation site and its modification affects the ATP-binding and *oriC*-binding abilities of DnaA *in vitro*. Our findings suggest that acetylation is involved in the DNA replication initiation and provide a novel mechanism for regulating the activity of DnaA in bacteria.

## Results

### DnaA is dissociated from *oriC* at the stationary phase

**Figure 1:**
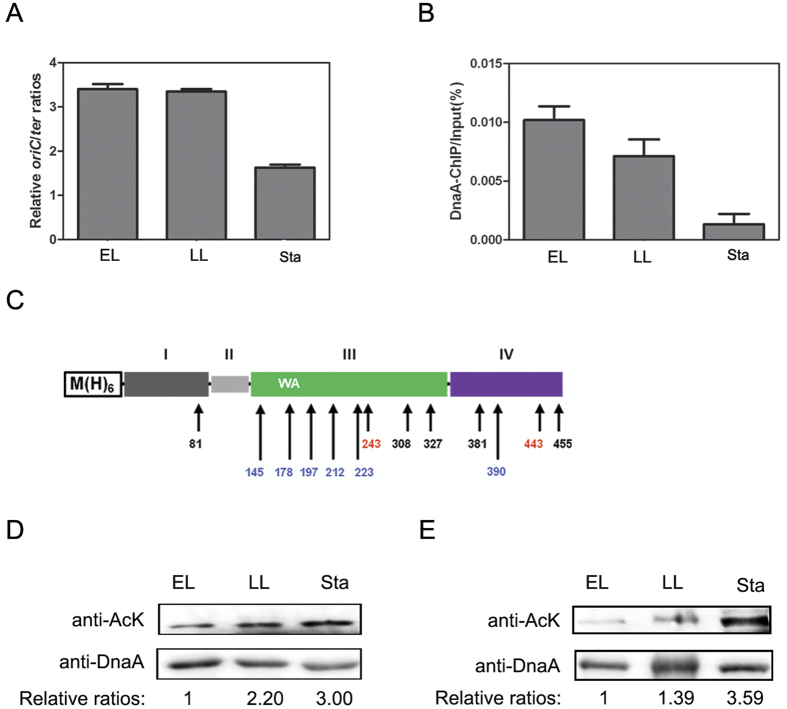
Analysis of DnaA acetylation *in vivo*. (**A**) Cellular levels of *oriC,* and *ter* were quantified. *E. coli* strain BL21 were cultured in LB medium at 37 °C and collected at early logarithmic (EL) phase (OD_600_ ~ 0.3), late logarithmic (LL) phase (OD_600_ ~ 1.2) and stationary phase (OD_600_ ~ 4.0). The genomic DNA was extracted from the cells and *oriC/ter* ratios were determined by qPCR, Error bars represented SD from three independent experiments. (**B**) DnaA-ChIP of *oriC*. *E. coli* strain BL21 cells at different growth phase were collected, and the DNA fragments bound to DnaA were immunoprecipitated according to the ChIP assay presented in the Supplementary methods. The relative *oriC* levels before (Input) and after (DnaA-ChIP) IP using DnaA antibody were determined using qPCR, yielding the ChIP*/*Input ratio (expressed as %). Error bars represented SD from three independent experiments. (**C**) The schematic plot of chromosome knock-in strain (His-DnaA) and acetylation sites of natively expressed DnaA. *E. coli* strain BL21-his-dnaA cells were cultured in LB medium at 37 °C and were collected at EL phase (OD_600_ ~ 0.3), late LL)phase (OD_600_ ~ 1.2) and stationary phase (OD_600_ ~ 4.0). The natively expressed DnaA were purified and were analyzed by mass spectrometry. The unique acetylated lysine residues of DnaA at the EL or stationary phase were labeled in red or blue, respectively, and shared lysine residues by both phases were labeled in black. (**D**) Acetylation patterns of DnaA from *E. coli* strain BL21 at the EL phase, LL phase and stationary phase (Sta). DnaA proteins were resolved on 10% SDS-PAGE and probed with DnaA antibody and acetylated-lysine antibody (AcK). The relative ratios are referred to anti-AcK:anti-DnaA ratios. Western blots are representative from at least three independent replicates. (**E**) Acetylation patterns of DnaA from *E. coli* strain BW25113 at the EL phase, LL phase and stationary phase (Sta). DnaA proteins from *E. coli* strain BW25113 at the EL phase, LL phase and stationary phase in were resolved on 10% SDS-PAGE and probed with DnaA antibody and acetylated-lysine antibody (AcK). The relative ratios are referred to anti-AcK:anti-DnaA ratios. Western blots are representative from at least three independent replicates.

Bacterial chromosomal replication initiates at *oriC* and proceeds bidirectionally to *ter* located on the opposite side of the circular chromosome. Generally, during fast growth period, it takes longer time to complete chromosome replication than the generation time; therefore, initiation occurs more than once on partially replicated chromosomes, and cells contain multiple replication forks. The cellular DNA replication initiation frequency can be denoted by the ratio of *oriC*/*ter*. To examine the initiation frequency in different growth phases, we detected the *oriC*/*ter* ratios of cells at the early logarithmic (EL), late logarithmic (LL) and stationary (Sta) phases. Figure 1A showed that the *oriC*/*ter* ratios at the early logarithmic (EL), late logarithmic (LL) were about 2.05-fold and 2.01-fold respectively to that at the stationary phase, indicating that bacterial cells have low initiation frequency at the stationary phase.

At the stationary phase, bacterial cells grow slowly and the inter-initiation time is longer than that in other phases^9^,^10^,^11^. The binding of DnaA to *oriC* is the prerequisite to form the efficient initiation complex. Therefore, we performed ChIP (Chromatin immunoprecipitation) assay to investigate whether DnaA binding to *oriC* is dependent on growth phase. *E. coli* strain BL21 cells with an initial OD_600_ ~ 0.1 were cultured in LB medium at 37 °C. We collected cells at early logarithmic (EL) phase (OD_600_ ~ 0.3), late logarithmic (LL) phase (OD_600_ ~ 1.2), stationary phase (OD_600_ ~ 4.0) and immunoprecipitated DNA-protein complexes from these cells by using DnaA antibody. The enriched *oriC* was quantified by real-time quantitative PCR (qPCR). The ChIP assay showed that the association between DnaA and *oriC* continuously reduced from early logarithmic (EL) phase to stationary phase, especially at the stationary phase (Fig. 1B). These results revealed that DnaA bound to *oriC* in a growth phase-dependent manner and dissociated from *oriC* dramatically at the stationary phase, which may partially explain why the inter-initiation time is protracted at the stationary phase. Decrease in *oriC*-DnaA binding can be explained by decrease of the ATP-DnaA level. Thus, ADP-DnaA-*oriC* complexes rather than ATP-DnaA-*oriC* complexes are predominantly formed. ADP-DnaA-*oriC* complexes are less stable than ATP-DnaA-*oriC* complexes, which would decrease the recovery of *oriC* by ChIP. However, at the stationary phase, the cell cycle-coordinated RIDA and DDAH fail to decrease ATP-DnaA level. It seems that only the inactivation of DARSs is insufficient for maintaining ATP-DnaA at a lower level and suggests there may be other independent mechanisms to maintain the lower level ATP-DnaA at the stationary phase.

### The acetylation level of natively expressed DnaA varies with growth phases

Our previous study demonstrated that protein acetylation is extensively distributed in *Salmonella*^16^. Since then, we further identified more acetylated proteins including DnaA in *E. coli*. Interestingly, Schilling and his coworkers also found DnaA was acetylated in *E. coli*^21^. Considering the fact that the binding of DnaA to *oriC* varied with growth phases (Fig. 1B), we hypothesized that the acetylation modification of DnaA may vary in different conditions and lead to the change of its binding affinities to *oriC*. To test this speculation, we knocked in a 6XHis-tag nucleotide sequence at N terminus of *dnaA* in *E. coli* chromosome (Fig. 1C). It was reported that the activity of polyhistidine-tagged DnaA protein was comparable to non-tagged DnaA protein^32^. By using this strategy and Nickel-column affinity purification, the natively expressed DnaA proteins were isolated from various genetic backgrounds and different growth stages. For the purification of the natively expressed DnaA proteins from wild-type (WT) strain, we collected cells at early logarithmic (EL) phase, late logarithmic (LL) phase, and stationary phase. Strikingly, DnaA proteins from *E. coli* strain BL21 at the stationary phase showed higher acetylation level compared with proteins from early logarithmic (EL) or late logarithmic (LL) phase. The acetylation level of DnaA at the stationary phase was about 3-fold to that of DnaA at the early logarithmic (EL) phase (Fig. 1D). Moreover, this phenomenon was not strain-specific since DnaA proteins from *E. coli* K strain BW25113 also showed similar acetylation trend (Fig. 1E).

### The reversible acetylation of DnaA is dependent on CobB, YfiQ and AcP *in vitro* and *in vivo*

**Figure 2:**
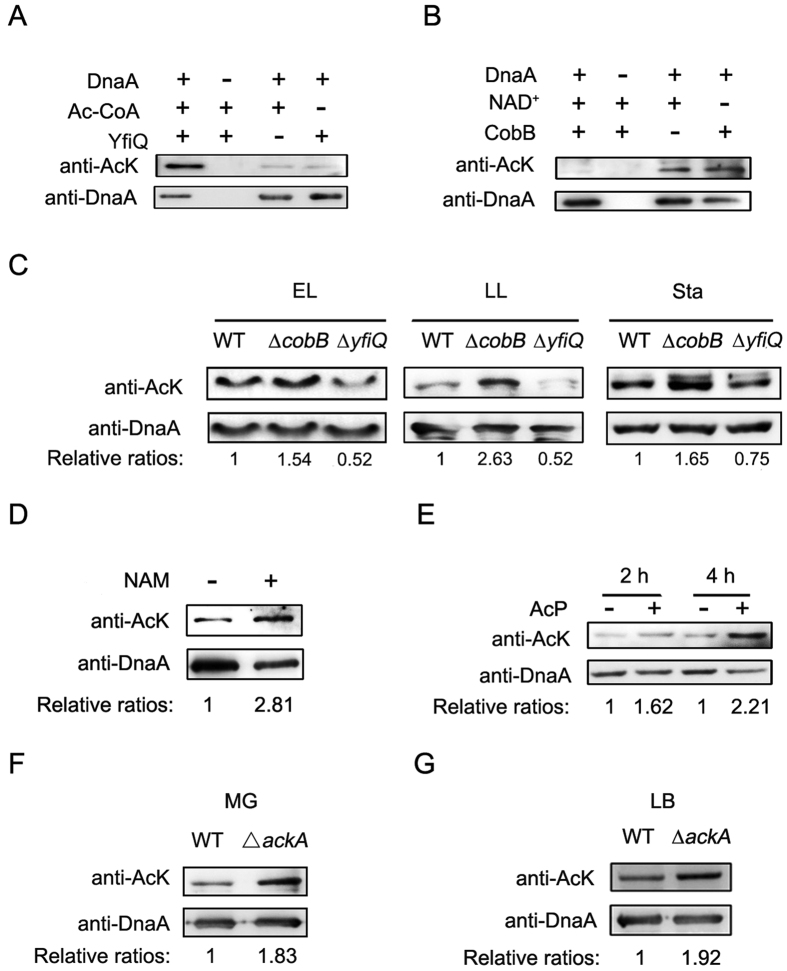
The acetylation of DnaA *in vitro* and *in vivo*. **(A**) YfiQ acetylates DnaA *in vitro*. DnaA (300 ng) was purified and incubated with or without YfiQ (50 ng). Products were resolved on 10% SDS-PAGE and probed with DnaA antibody and acetylated-lysine antibody (AcK). Western blots are representative from at least two independent replicates. (**B**) CobB deacetylates DnaA *in vitro*. DnaA (300 ng) from stationary phase of ∆*cobB* was purified and incubated with or without CobB (50 ng). Products were resolved on 10% SDS-PAGE and probed with DnaA antibody and acetylated-lysine antibody (AcK). Western blots are representative from at least two independent replicates. (**C**) The acetylation of DnaA is regulated by CobB and YfiQ. DnaA proteins from *E. coli* strain BL21, ∆*cobB* or ∆*yfiQ* at the early logarithmic (EL), late logarithmic (LL) or stationary phase were purified and resolved on 10% SDS-PAGE followed by Western blot analysis using DnaA antibody and acetylated-lysine antibody (AcK). Western blots are representative from at least three independent replicates. (**D**) The physiological acetylation level of DnaA increased when cells were grown in the presence of NAM. The DnaA proteins purified from *E. coli* strain BL21 grown in LB containing 10 mM NAM to an OD_600_ of 2.0 at 37 °C were analyzed by Western blot using DnaA antibody and acetylated-lysine antibody. Western blots were independently repeated at least three times. (**E**) AcP directly acetylates DnaA *in vitro*. DnaA (300 ng) was incubated with or without acetyl-phosphate (AcP) (10 mM) for 2 h or 4 h. Products were resolved on 10% SDS-PAGE and probed with DnaA antibody and acetylated-lysine antibody (AcK). Western blots were independently repeated at least three times. (**F,G**) The acetylation of DnaA was increased by the elevation of AcP synthesis. DnaA proteins from *E. coli* strain BL21 and ∆*ackA* at the logarithmic phase in MG medium (**F**) or LB medium (**G**) were purified and resolved on 10% SDS-PAGE and probed with DnaA antibody and acetylated-lysine antibody (AcK). The relative ratios are referred to anti-AcK: anti-DnaA ratios. Western blots were independently repeated at least three times.

Since the acetylation of DnaA fluctuated with growth phases, we next tried to identify the factors regulating the acetylation status of DnaA. It has been found that lysine acetyltransferase YfiQ and deacetylase CobB are responsible for the acetylation and deacetylation of multiple proteins^26^,^33^,^34^. Therefore, we tested whether YfiQ and CobB were involved in the acetylation of DnaA. The purified YfiQ and DnaA were incubated for 3 h in the presence of Ac-CoA, and Western blot showed the acetylation level of DnaA increased dramatically after incubation with YfiQ compared with controls (Fig. 2A). To examine whether DnaA is a substrate of CobB, the natively expressed DnaA from the deletion mutant of *cobB* at the stationary phase was treated by CobB at the presence of NAD^+^, and Western blot showed that CobB can deacetylate DnaA *in vitro (*Fig. 2B).

To test whether YfiQ and CobB is involved in the modification of DnaA *in vivo*, we constructed chromosomal His-tag knock-in strains (His-DnaA) in the deletion of *yfiQ* or *cobB* background. DnaA proteins were purified from ∆*yfiQ* or ∆*cobB* strains as well as wild-type strain at different growth phases and applied to Western blot analysis. Figure 2C showed the acetylation level of DnaA increased when *cobB* was deleted, while decreased when *yfiQ* was deleted regardless of cell growth phases. These results indicated that YfiQ and CobB were involved in the acetylation and deacetylation of DnaA *in vivo*, respectively. Consistent with the aforesaid observations, treatment of wild-type cells with NAM (nicotinamide)^35^, an inhibitor for CobB, caused the increase of acetylation of natively expressed DnaA (Fig. 2D).

It has been reported that AcP (acetyl-phosphate) could acetylate proteins nonenzymatically and played a critical role in protein acetylation in *E. coli*^18^,^19^. To test whether AcP is involved in the acetylation of DnaA, we performed AcP acetylation assay *in vitro*. The acetylation level of DnaA protein was dramatically increased after AcP treatment in a time-dependent manner (Fig. 2E). Based on this finding, we created the deletion mutant of *ackA* (acetate kinase), whose cellular AcP was accumulated^36^,^37^,^38^,^39^, and isolated natively expressed DnaA protein from this strain at the logarithmic phase in the M9 supplemented with glucose (MG) media, which can increase the synthesis of AcP^38^,^40^. Western blot showed that the acetylation level of DnaA was higher in the deletion mutant of *ackA* compared with the control strain (Fig. 2F). This phenomenon was also confirmed when LB medium were used (Fig. 2G). Therefore, the above findings domenstrated that YfiQ, AcP and CobB were responsible for the acetylation and deacetylation of DnaA both *in vitro* and *in vivo*, respectively.

### The key K178 residue of DnaA is acetylated

To identify the acetylated lysine residues of DnaA, the purified DnaA proteins from wild-type strain at the early logarithmic (EL) or stationary phase were analyzed by mass spectrometry. Seven acetylated lysine residues were detected, including K81, K243, K308, K327, K381, K443 and K455 in DnaA from early logarithmic (EL) phase and 11 lysine residues were acetylated from stationary phase DnaA including K81, K145, K178, K197, K212, K223, K308, K327, K381, K390 and K455. All the acetylated lysine residues identified by mass spectrometry were illustrated in Fig. 1C by color. We identified more acetylated lysine residues of DnaA at the stationary phase than early logarithmic (EL) phase, which was consistent with the increase of acetylation level from early logarithmic (EL) phase to stationary phase (Fig. 1D). 12 of 13 acetylated lysine residues were located in the Domain III or Domain IV of DnaA. Since Domain III and Domain IV are responsible for ATP- and DNA-binding, respectively^41^, we speculate that acetylation of lysine residues in those regions may be involved in regulating DnaA activities.

We next performed a plasmid complementation test in *E. coli* strain KA413 to evaluate the function of all the above acetylated lysine residues in DNA replication^42^. *E. coli* strain KA413 was a temperature-sensitive *dnaA*46 mutant, and DnaA46 protein was unstable at 42 °C and caused failure of bacterial growth^43^. A total of 16 lysine residues (besides the 13 acetylated lysine residues mentioned above, we also included K135, K309 and K397 as well, though not detected as acetylated by mass spectrometry) were mutated individually to arginine (R) or glutamine (Q) by site-directed mutagenesis to evaluate their importance. Plasmids bearing the above *dnaA* mutations were transformed into *E. coli* strain KA413. For most lysine mutations, colonies were formed at 42 °C or 30 °C with a similar efficiency (Supplementary Table S3). Interestingly, when K178 was mutated to glutamine (K178Q) or arginine (K178R), the transformed host strain failed to form any colonies at 42 °C (Table 1). We speculated that the mutation K178Q by changing the charge of its side chain and the mutation K178R by disrupting its structure interfered the activity of DnaA. These results highly suggest that K178 is essential and plays a critical role in regulating DNA replication initiation.

### An intact lysine at residue 178 is required for the activities of DnaA

**Figure 3:**
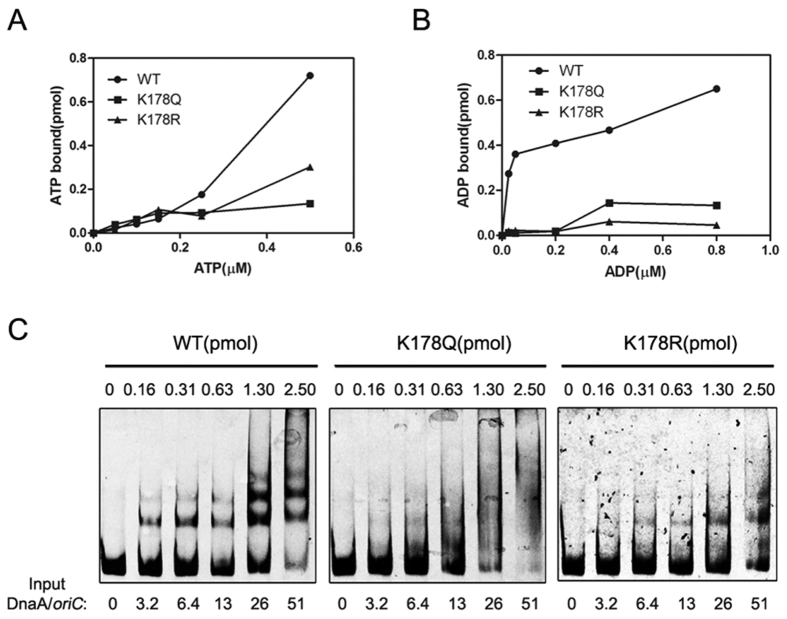
An intact lysine at residue 178 is required for the activities of DnaA. (**A**,**B**) DnaA binding affinity to ATP or ADP. The binding abilities of DnaA and its derivatives were measured by filter-binding assay. DnaA K178Q (solid square) and DnaA K178R (solid triangle) and the wild-type DnaA protein (solid cycle) was incubated with various concentrations of [α-^32^P]ATP (A) or [2, 8-^3^H]ADP (**B**) for 15 min at 0 °C. The amount of bound ATP or ADP was determined by scintillation. The results are representative from at least three independent replicates. (**C**) DnaA K178Q and K178R form fewer multimers on *oriC*. The indicated amounts of wild-type DnaA and DnaA K178Q and K178R were incubated for 5 min at 20 °C with a 5′-FAM-*oriC* fragment (469 bp, 7 ng) in the presence of 2 mM ATP. Reaction products were analyzed by 5% native PAGE electrophoresis. The blots are representative from at least three independent replicates.

K178 is located in the Walker A motif (GXXGXGKT/S) of DnaA Domain III and highly conserved in bacteria, archaea and eukaryotic cells^44^. Since Walker A motif of DnaA is involved in ATP/ADP and *oriC* binding, we next examined whether the mutation of K178 affects its ATP- or *oriC*-binding abilities. Although K178 was shown to be critical for the function of DnaA in *E. coli*^45^, the underlying mechanism is still unknown. The wild-type DnaA, DnaA (K178Q) and DnaA (K178R) were overexpressed and purified to apparent homogeneity (Supplementary Fig. S1). A filter retention assay indicated that K178Q and K178R lost their binding abilities to ATP compared with the wild-type DnaA (Fig. 3A). Moreover, mutation of K178 completely abolished the ability of DnaA to bind ADP (Fig. 3B). The *oriC* binding assay showed the wild-type DnaA can form complexes with *oriC* at a concentration as low as 0.16 pmol, whereas neither K178Q nor K178R formed complex at this concentration (Fig. 3C), which suggests that K178 residue is indispensable for DnaA to bind with ATP/ADP and *oriC*.

### Acetylation of K178 inhibits activities of DnaA

**Figure 4:**
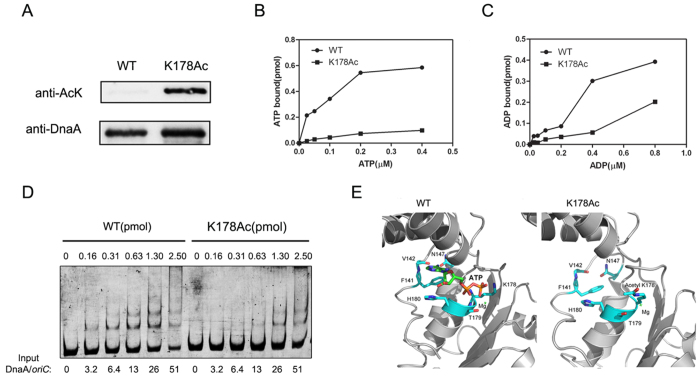
Acetylation of K178 inhibits the activities of DnaA. (**A**) Western blot analysis of DnaA K178Ac. DnaA K178Ac was produced in the presence of pAcKRS-3/pCDF-PylT-*dnaA* (K178TAG) with 10 mM εN-acetyllysine (AcK) and purified by inclusion body renaturation. The antibodies are acetylated-lysine antibody (AcK) and DnaA antibody. Wild-type DnaA proteins were overexpressed and purified from *E. coli* strain BL21. (**B**,**C**) The abilities of DnaA K178Ac binding of ATP and ADP were measured by filter-binding assay. DnaA K178Ac (solid square) and the wild-type DnaA protein (solid cycle) was incubated with various concentrations of [α-^32^P]ATP (**B**) or [2, 8-^3^H]ADP (**C**) for 15 min at 0 °C. The amount of bound ATP or ADP was determined by filter-binding assay. The results are representative from at least three independent replicates. (**D**) DnaA K178Ac forms fewer multimers on *oriC*. The indicated amounts of wild-type DnaA and DnaA K178Ac were incubated for 5 min at 20 °C with a 5′-FAM-*oriC* fragment in the presence of 2 mM ATP. Reaction products were analyzed by 5% native PAGE electrophoresis. The blots are representative from at least three independent replicates. (**E**) The structure of the *E. coli* DnaA was modeled by Modeller 9.11 using the crystal structure of 1L8Q from RSC Protein Data Bank as its template. The acetyl K178 was modified and further optimized using the Sybyl 6.8 program. Data showed ATP combined into the substrate pocket and the surrounding residues in the homology structure of the *E. coli* DnaA in the wild-type condition, and acetylated K178 had a steric clash with the γ-phosphate group of ATP.

Then, we used a site-specifically incorporated εN-acetyllysine system to obtain K178-specific acetylated DnaA^46^. Briefly, εN-acetyllysine was incorporated into recombinant protein with high translational fidelity and efficiency in response to the amber codon, *via* an orthogonal εN-acetyllysyl-tRNA synthetase/tRNA (MbPylRS/MbtRNA_CUA_) pair evolved from methanogenic *Methanosarcina barkeri*. This system was used successfully in multiple studies to investigate the roles of acetylation on protein functions^47^,^48^,^49^. The DnaA (K178Ac) protein was purified to apparent homogeneity (Supplementary Fig. S2), and incorporation of acetyllysine was verified by Western blot (Fig. 4A). The filter retention assay showed the ATP- or ADP-binding abilities decreased dramatically after K178 was acetylated (Fig. 4B,C). The *oriC* binding assay showed DnaA (K178Ac) formed complexes with *oriC* at a concentration of 0.63 pmol, which was 4-fold higher than wild-type DnaA (Fig. 4D).

### Crystal structure modeling demonstrates acetylation of K178 of DnaA might prevent ATP from binding

Since the crystal structure of domain III and VI of DnaA from *Aquifex aeolicus* has been resolved^50^ and this fragment shares 40% identity and 60% similarity with that of DnaA from *E. coli*, we used Modeller 9.11 to model the structure of the *E. coli* DnaA. The modeling data showed that the terminal nitrogen atom of K178, the counterpart of K125 in DnaA of *A. aeolicus*, stabilized ATP by formulating an H-bond with its phosphate group. When the K178 was acetylated, the modified side-chain flipped to the substrate pocket and had a steric hindrance on the phosphate group of ATP, leading to the failure to bind ATP (Fig. 4E). Surface representation model clearly showed that ATP pocket was intact in the wild-type protein, while acetyl group of K178Ac blocked ATP binding (Supplementary Fig. S3). Therefore, we conclude that the acetylation of K178 prevented DnaA from binding to ATP/ADP and subsequently undermined its binding to the chromosomal origin *oriC* and replication initiation as well.

### CobB is responsible for the deacetylation of K178, YfiQ and AcP are responsible for the acetylation K178 *in vitro*

**Figure 5:**
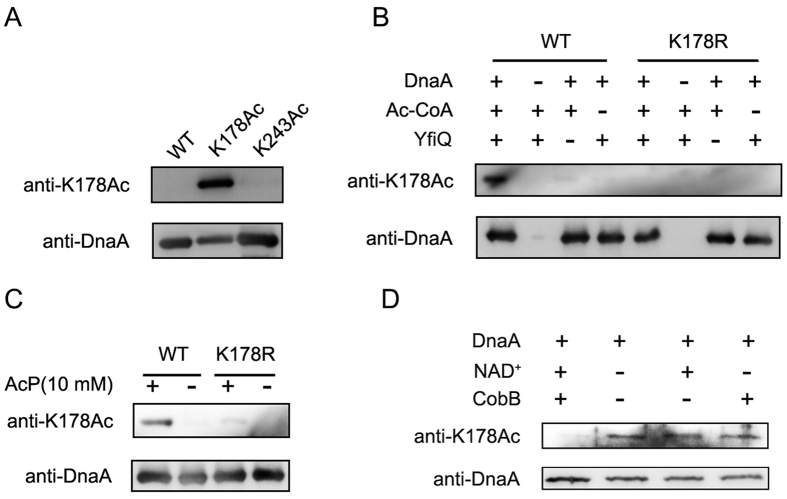
The acetylation of K178 *in vitro*. (**A**) The evaluation of the specificity and sensitivity of K178-specific acetylated DnaA (K178Ac) antibody. Wild-type DnaA protein (WT), DnaA (K178Ac) and K243-specific acetylated DnaA (K243Ac) were resolved on 10% SDS-PAGE and probed with DnaA antibody or DnaA (K178Ac) antibody. Western blots were independently repeated at least three times. Wild-type DnaA proteins were overexpressed and purified from *E. coli* strain BL21. (**B**) YfiQ acetylates DnaA *in vitro*. DnaA (300 ng) and DnaA (K178R) were purified and incubated with or without YfiQ (50 ng). Products were resolved on 10% SDS-PAGE and probed with DnaA antibody and DnaA (K178Ac) antibody. Western blots were independently repeated at least three times. (**C**) AcP directly acetylates K178 of DnaA *in vitro*. DnaA and DnaA (K178R) proteins were incubated with or without acetyl-phosphate (AcP) (10 mM) for 4 h. Products were resolved on 10% SDS-PAGE and probed with DnaA antibody and DnaA (K178Ac) antibody. Western blots were independently repeated at least three times. (**D**) CobB deacetylates K178Ac of DnaA *in vitro*. DnaA (K178Ac) was incubated with or without CobB (50 ng). Products were resolved on 10% SDS-PAGE and probed with DnaA antibody and DnaA (K178Ac) antibody. Western blots were independently repeated at least three times.

Since the acetylation of K178 is critical for the regulation of DnaA activities, we want to examine whether the above-mentioned acetylation and deacetylation agents, CobB, YfiQ and AcP, were involved the modification of this specific residue. We firstly prepared the site specific anti-K178Ac antibody, and the specificity and sensitivity of the anti-K178Ac antibody were verified by Western blot (Fig. 5A). The wild-type DnaA and the mutant DnaA (K178R) were purified and treated by YfiQ or AcP followed by Western blot analysis with DnaA (K178Ac) antibody. After treatment with YfiQ, the acetylation level of K178 of wild-type DnaA increased dramatically (Fig. 5B). Instead, no anti-K178Ac signal could be detected on DnaA (K178R), further highlighting the specificity of this anti-K178Ac antibody. AcP incubation showed similar result as YfiQ treatment (Fig. 5C). Then we purified K178-specific acetylated DnaA and treated it with CobB, and Western blot showed that CobB could remove the acetyl group of DnaA-K178 completely at the presence of NAD^+^ (Fig. 5D). These data demonstrated that both YfiQ and AcP can acetylate K178 of DnaA, while CobB can deacetylate K178 of DnaA *in vitro*, respectively.

### CobB is responsible for the deacetylation of K178, YfiQ and AcP are responsible for the acetylation of K178 *in vivo*

**Figure 6:**
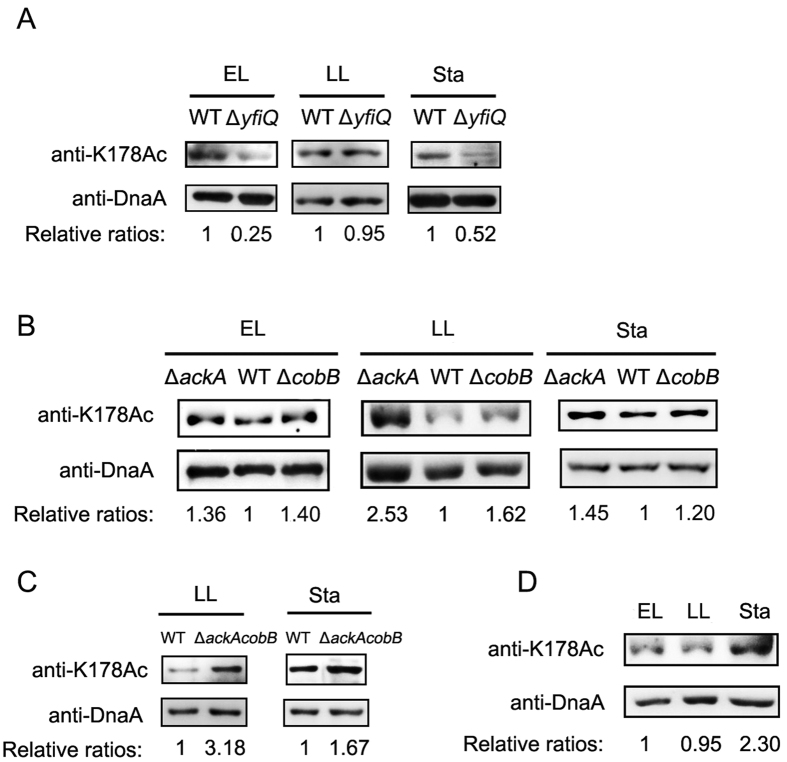
K178 acetylation level was regulated in a growth phase–coordinated manner. (**A**) K178Ac of DnaA is regulated by YfiQ *in vivo*. DnaA proteins from wild-type strain and ∆*yfiQ* cultured in LB medium at 37 °C at the early logarithmic (EL), late logarithmic (LL) or stationary phase were purified and resolved on 10% SDS-PAGE and probed with DnaA antibody and DnaA (K178Ac) antibody. The relative ratios are referred to anti-AcK:anti-DnaA ratios. Western blots were independently repeated at least three times. (**B,C**) K178Ac of DnaA is regulated by CobB and AcP *in vivo*. DnaA from wild-type strain, ∆*cobB* or ∆*ackA* or ∆*ackAcobB* at the early logarithmic (EL), late logarithmic (LL) or stationary phase were purified and resolved on 10% SDS-PAGE and probed with DnaA antibody and DnaA (K178Ac) antibody. The relative ratios are referred to anti-AcK:anti-DnaA ratios. Western blots were independently repeated at least three times. (**D**) Acetylation pattern of K178 in DnaA from *E. coli* wild-type strain cultured in LB medium at 37 °C at the early logarithmic (EL), late logarithmic (LL) and stationary phase (Sta). DnaA proteins were purified and resolved on 10% SDS-PAGE and probed with DnaA antibody and DnaA (K178Ac) antibody. The relative ratios are referred to anti-AcK: anti-DnaA ratios. Western blots were independently repeated at least three times.

To further confirm whether YfiQ, AcP and CobB were responsible for the modification of K178 of DnaA *in vivo*, we analyzed the acetylation level of DnaA with anti-K178Ac antibody. DnaA proteins from the deletion mutants of *yfiQ*, *ackA* and *cobB* cultured in LB medium at different growth phases were purified and analyzed by Western blot. Figure 6A showed that the acetylation of K178 of DnaA from *∆yfiQ* decreased dramatically compared with that of wild-type at the stationary phase. DnaA from the deletion mutant of *ackA* showed higher K178 acetylation level compared with that of the wild-type strain (Fig. 6B). The acetylation levels of K178 of *∆ackA* were about 2.5- and 1.45-fold to these of wild-type strains at the late logarithmic (LL) phase and stationary phase, respectively. The acetylation levels of K178 in *∆cobB* were about 1.4- and 1.6-fold higher than these of wild-type strains at the early logarithmic (EL) and late logarithmic (LL) phase, respectively (Fig. 6B). At the stationary phase, the acetylation level of K178 in *∆cobB* was just a little higher than that of the wild-type strain. To further confirm the contribution of AcP and CobB in regulating the acetylation level of DnaA K178 *in vivo*, we investigated the acetylation level of K178 in *ackA* and *cobB* double deletion mutant. As shown in Fig. 6C, no matter at late logarithmic phase or at stationary phase, the acetylation levels of K178 in *∆ackAcobB* double mutant were higher than those in *∆ackA* (Fig. 6C). The above data suggested that both YfiQ and AcP contributed to acetylation of DnaA K178 *in vivo* and CobB was involved in deacetylation of DnaA K178Ac in a growth phase-dependent manner.

### The acetylation level of K178 is elevated dramatically at the stationary phase

We have already shown that the overall acetylation status of DnaA correlated with cell growth and DNA replication initiation frequencies, since K178 is the most critical residue in maintaining the activities of DnaA, we want to test whether the acetylation level of K178 parallels with the overall acetylation trend. Western blot analysis showed that DnaA proteins from stationary phase showed higher acetylation level of K178 compared with proteins from early logarithmic (EL) or late logarithmic (LL) phases (Fig. 6D). The acetylation level at K178 of DnaA at the stationary phase was about 2.3-fold higher than that of DnaA at the early logarithmic (EL) phase. These data showed that the acetylation level of K178 was associated with growth phase and culminated at the stationary phase, which was consistent with the global DnaA acetylation pattern (Fig. 1D).

## Discussion

DNA replication initiation is tightly controlled and must occur at right time. It is well demonstrated that DnaA is the major controller of replication initiation, which is dependent on enough accumulation of ATP-DnaA. Although several regulatory pathways for DNA replication initiation have been identified and well documented in *E. coli*, these pathways are all in a cell cycle-coordinated manner^7^,^8^,^51^,^52^. At the stationary phase, bacterial cells become smaller and grow slowly and the inter-initiation time is prolonged^9^,^10^,^11^,^34^. Whether and how the cells coordinate their growth phase with replication initiation frequencies at post-replicational level remain elusive.

We revealed that the binding ability of DnaA to *oriC* decreased dramatically at the stationary phase (Fig. 1B). Since the cellular DnaA content maintained a relatively stable level in all growth phases^12^, we speculate that the decrease of DnaA binding at *oriC* region is not due to the shortage of DnaA protein, but rather a certain change of DnaA, leading to its failure to bind *oriC* at the stationary phase. It is known that PTM could change protein properties, we thus sought to detect the modification of DnaA by mass spectrometry. We showed that the acetylation level of DnaA increased in a growth phase-dependent manner and peaked at the stationary phase (Fig. 1D,E). A recent paper by Schilling and co-workers described the increasing acetylation level of hundreds of proteins in response to carbon overflow in *E. coli*^21^. It seems that the increasing acetylation level of proteins at stationary is beneficial for bacterial survival. We propose that the acetylation of DnaA is involved in the DnaA dissociating from *oriC* at the stationary phase and is a response mechanism for bacteria encountering stressful conditions.

In this work, we identified 13 acetylated lysine residues in total in natively expressed DnaA from the wild-type strain. Plasmid complementation assay showed that K178 is a key acetylated lysine site and plays a critical role in the function of DnaA (Table 1). It has been reported that K178 is required for DnaA binding to ATP or ADP^45^. Our data showed that both DnaA (K178Q) and DnaA (K178R) lost the binding ability to ATP or ADP (Fig. 3A,B). Furthermore, the acetylation of K178 decreased dramatically the binding abilities of DnaA to ATP or ADP (Fig. 4B,C). It was known that the conserved lysine residue in the Walker A motif, together with the main chain NH atoms, are critical for nucleotide-binding^53^. In fact, the ε-NH_3_^+^ of the lysine residue possesses positive charge and interacts with the negatively charged phosphate group of ATP to stabilize ATP binding. While the lysine residue was acetylated, the protein lost the positive charge of the lysine residue ε-NH_3_^+^ (positive change of ε-NH_3_^+^ group on lysine residue), and consequently caused disassociation of ATP from protein. Combined with the surface representation model (Supplementary Fig. S3), we conclude that acetylation disrupted the integrity of K178 side chain and blocked DnaA binding to ATP, and then decreased DnaA binding ability to *oriC*.

YfiQ-dependent and AcP-dependent acetylation are the two known primary mechanisms for lysine acetylation^18^,^19^,^33^,^54^. We found that both YfiQ and AcP are responsible for DnaA acetylation in *E. coli*. To determine whether YfiQ and AcP cooperate with each other in the acetylation of DnaA, we performed qPCR to analyze mRNA levels of *yfiQ* at different phases. Our data showed that the transcriptional level of *yfiQ* at the late logarithmic phase was highest (Supplementary Fig. S4A). The concentration of AcP was more than 10-fold-increased in *E. coli* cells at the stationary phase compared with that at logarithmic phase^21^,^38^,^40^,^55^. It seems that YfiQ mainly contributed to the acetylation of DnaA during the transition from the late logarithmic phase to the stationary phase, and AcP played a major role in the acetylation of DnaA at the stationary phase. CobB is the predominant deacetylase that shows no preference for enzymatic and nonenzymatic lysine acetylation substrate sites in *E. coli*^27^. The transcriptional level of *cobB* decreased at the stationary phase (Supplementary Fig. S4B), which might increase the acetylation level of DnaA synergistically with AcP to coordinate the cell growth rate.

**Figure 7:**
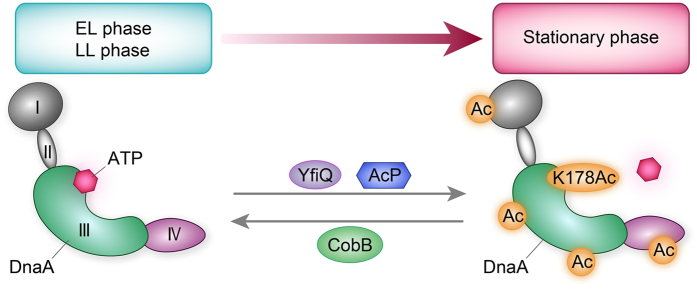
A model of the reversible acetylation of DnaA in regulating DNA replication initiation. The acetylation level of DnaA varied with the cell growth. At the early logarithmic (EL) and late logarithmic (LL) phases, the DnaA acetylation level is low, and DnaA binding ability to ATP and *oriC* is high, which results in the rapid replication initiation and cell growth rate. At the stationary phase, DnaA acetylation level especially the site of K178Ac reaches the summit and DnaA binding ability to ATP and *oriC* decreases seriously, coordinates the slow growth rate and low DNA replication initiation frequencies. During this process, CobB, YfiQ and AcP play crucial roles in dynamically deacetylating and acetylating DnaA to coordinate the cell growth phase.

Our data showed that the reversible acetylation of K178 were regulated by CobB, YfiQ and AcP and the acetylation level of K178 was increasing in a growth phase dependent manner, culminating at the stationary phase, which was consistent with the global DnaA acetylation pattern (Fig. 1D,5 and 6). Combined with the aforesaid results that acetylation of K178 weakened the activities of DnaA, and DnaA dissociated dramatically from *oriC* at the stationary phase (Fig. 1B), we propose a working model to explain how lysine acetylation of DnaA coordinates DNA replication initiation (Fig. 7). In this model, the acetylation level of DnaA is low in early logarithmic (EL) and late logarithmic (LL) phases, and DnaA binding abilities to ATP and *oriC* are relatively high, which coordinates the rapid replication initiation and cell growth. However, the acetylation level of DnaA, especially at the site of K178, reaches the summit at the stationary phase. The decreasing amount of DnaA-ATP leads to the decreasing ratio of DnaA-ATP/DnaA-ADP and affects DnaA-*oriC* interaction at low affinity recognition sites to cause the unwound of *oriC* in stationary phase. Those finally results in the low DNA replication initiation frequency and slow growth. During the reversible acetylation process, CobB, YfiQ and AcP play crucial roles in dynamically deacetylating and acetylating DnaA respectively to coordinate the replication initiation frequencies and cell growth. Among the 13 acetylated lysine residues examined in this study, we found K178 plays a major role in regulating the activities of DnaA. It is possible that other lysine residues may play important roles in regulating the activities of DnaA by reversible acetylation, which requires further investigations in the future.

The present work provides an example in which lysine acetylation regulates protein activity in bacteria. In this study, we showed that K178 located in Walker A motif is a key acetylation site by inhibiting the ATP- and *oriC*-binding abilities of DnaA. Since all the DNA replication initiators in archaea, bacteria and eukaryotic cells possess Walker A motif^44^, we speculate that the regulation of DNA replication by acetylation of Walker A motif could be a conserved and universal mechanism to control DNA replication in various organisms. Furthermore, Walker A motif is also shared among a variety of proteins with important functions, including AAA + proteins, ATP synthases, helicases, kinases, ABC transporters and G-proteins^44^. The conserved lysine residue of the Walker A motif is crucial for nucleotide-binding^53^. Therefore, we propose that lysine acetylation might be a general regulation strategy to control various cellular processes by fine-tuning the activities of ATP-requiring proteins in response to environmental stimuli.

## Methods

Detailed information about experimental procedures not provided here can be found in the Supplementary methods.

### Bacterial strains, plasmids, primers and media

All strains, plasmids and primers used in this study are described in Supplementary Table S1 and Table S2. Luria-Bertani broth (LB) was used as rich medium, nutrient agar plates contained 1.5% (W/V) agar and supplemented with antibiotics as required. The antibiotics used were 100 μg/ml of ampicillin, 17 μg/ml of chloramphenicol, 50 μg/ml of kanamycin and 50 μg /ml of spectinomycin.

### Generation of chromosomal knock-in strains

For the construction of *E. coli*-*his-dnaA*, taking advantage of fusion PCR technology and using His-DnaA F1, R1, F2, R2, F3, R3, F4, R4, PCR product containing a 6XHis-tag placed at the N-terminus of *dnaA* and a cm resistance cassette between *rpmH* 3p and *dnaA* 1p was amplified. PCR product was gel extracted, and electroporated into *E. coli* containing pKD46 prepared in the presence of arabinose. The knock-in strains were verified by PCR using knock-in check primers and sequencing of the PCR products.

### Plasmid complementation assay

To perform plasmid complementation assay, *E. coli* strain KA413 cells were transformed with pCDSSara bearing the indicated *dnaA* allele and incubated on LB agar plates containing thymine (50 μg/mL) and spectinomycin (100 μg/mL) at 30 °C for 24 h or on LB agar plates containing thymine (50 μg/mL), spectinomycin (100 μg/mL) and 10 mM Ara at 42 °C for 10 h. Transformation efficiencies and the ratios were calculated by colony counting.

### DnaA polyclonal antibody preparation

The DnaA protein tagged with hexahistidine (6XHis) was overexpressed using pET22b-*dnaA* in *E. coli* strain BL21. The tagged protein was purified by nickel affinity chromatography under 8 M urea denaturing conditions and used to immunize rabbits for the production of polyclonal antibodies.

### Anti-K178Ac of DnaA polyclonal antibody preparation

The immune peptide Ac-CGGTGLGK(Ac)THL-NH_2_ and the control peptide Ac-CGGTGLGKTHLLHA-NH_2_ were prepared for anti-K178Ac of DnaA antibody production. Briefly, the immune peptide was used as antigen to immunize mouse. During two months, mice were immunized for four times, and the antisera were collected and purified. The sensitivity and specificity of antibody were evaluated by ELISA and Western blot.

### ChIP assay

DNA fragments bound to DnaA were immunoprecipitated as described previously with some modifications^8^,^56^. Details are provided in the Supplementary methods.

### Western blot analysis

Western blot was carried out by following standard procedures. Details are provided in the Supplementary methods.

### Expression and purification of site-specific acetylated DnaA (K178Ac)

We expressed DnaA (K178Ac) in *E. coli* by genetically encoding the incorporation of εN-acetyllysine in response to the amber stop codon *via* the orthogonal εN-acetyl- lysyl-tRNA synthetase/tRNA_CUA_ pair^46^. Details are provided in the Supplementary methods.

### YfiQ-Mediated *In vitro* Acetylation

The *in vitro* reaction was performed as described previously^16^ with 300 ng DnaA in the absence or presence of 50 ng YfiQ. Reaction mixtures were incubated at 37 °C for 3 h and then resolved by 10% SDS-PAGE and analyzed by Western blot using anti-DnaA or anti-AcK antibody.

### CobB-Mediated *In vitro* Deacetylation

Deacetylation of DnaA and DnaA (K178Ac) *in vitro* was performed as described previously^57^. Briefly, 300 ng DnaA or DnaA (K178Ac) was incubated in 50 mM Tris-HCl buffer (pH 8.0) in the presence or absence of CobB at 37 °C for 3 h and then resolved by 10% SDS-PAGE and analyzed by Western blot using anti-DnaA or anti-AcK antibody.

### AcP-mediated *in vitro* acetylation

The AcP-mediated *in vitro* acetylation reaction was performed as described previously with some modification^18^. Briefly, DnaA was mixed with freshly prepared AcP at the final concentration of 10 mM (potassium lithium salt) at 37 °C for 2 h or 4 h and then resolved by 10% SDS-PAGE and analyzed by Western blot using anti-DnaA or anti-AcK antibody.

### NAM inhibition assay

Strain BL21-*his-dnaA* was grown in LB containing 10 mM NAM to an OD_600_ of 2.0 at 37 °C and collected. Purification of 6XHis-DnaA was carried out as described above. The DnaA from both NAM-treated cells and NAM-nontreated cells were purified and analyzed by Western blot using anti-DnaA or anti-AcK antibody.

### EMSA

EMSA (Electrophoretic Mobility Shift Assay) to test the interaction between DnaA and *oriC* was performed as described previously^42^, with some modifications. Details are provided in the Supplementary methods.

### Filter-binding assay

ATP- and ADP-binding activity of DnaA protein was determined by the filter-binding assay as described previously^58^. Details are provided in the Supplementary methods.

## Additional Information

**How to cite this article**: Zhang, Q. *et al*. Reversible lysine acetylation is involved in DNA replication initiation by regulating activities of initiator DnaA in *Escherichia coli*. *Sci. Rep.* **6**, 30837; doi: 10.1038/srep30837 (2016).

## Supplementary Material

Supplementary Information

## Figures and Tables

**Table 1:** K178 of DnaA is essential for DnaA activity.

Plasmid	Allele	Transformation efficiency (CFU/μg DNA)		
		30 °C	42 °C	42 °C/30 °C
pCDSSara*-dnaA*	*dnaA*	4.80*10^6^	5.60*10^6^	1.17
pCDSSara-*dnaA* (K178Q)	K178Q	9.70*10^7^	0	0
pCDSSara-*dnaA* (K178R)	K178R	4.86*10^6^	0	0
pCDSSara-vector	None	6.40*10^6^	0	0

*E. coli* strain KA413 was transformed with plasmid (250 ng) bearing the indicated *dnaA* allele and incubated on LB agar plates containing thymine (50 μg/ml) and spectinomycin (10 μg /ml) at 30 °C for 24 h or incubated on LB agar plates containing thymine (50 μg/ml) and spectinomycin (10 μg /ml) and 10 mM arabinose at 42 °C for 10 h. Transformation efficiencies and the ratios of 42 °C/30 °C were shown.
