# Supplementary material for: Reversible lysine acetylation is involved in DNA replication initiation by regulating activities of initiator DnaA in Escherichia coli
Source: Sci Rep. 2016 Aug 3;6:30837. doi: 10.1038/srep30837 (PMC4971506; doi:10.1038/srep30837)
Supplement: Supplementary Information [file srep30837-s1.pdf]

Reversible lysine acetylation is involved in DNA replication initiation by regulating activities of initiator DnaA in *Escherichia coli*

Qiufen Zhang[1], Aiping Zhou[1,2], Shuxian Li[1], Jinjing Ni[1], Jing Tao[1], Jie Lu[3], Baoshan Wan[1], Shuai Li[4], Jian Zhang[4], Shimin Zhao[5], Guo-Ping Zhao[6], Feng Shao[7], Yu-Feng Yao[1,2]*

Supplementary methods

Genetic Procedures

Gene cloning and plasmid construction

The genomic DNA of *E. coli* was extracted using Easy-DNA kit (Invitrogen, Carlsbad, CA). The genes mentioned in this manuscript were amplified with the corresponding primers listed in Table S2 using the *E. coli* MG1655 genomic DNA as template. The PCR products were digested with the corresponding restriction enzyme and then cloned into the vectors.

Generation of the deletion mutants of target genes

Mutants with deletions of the target genes including *yfiQ*, *cobB* and *ackA* mutant were constructed by the method described by Datsenko and Wanner[1]. Briefly, parental *E. coli* strain was transformed with the pKD46 vector, which contains genes coding for the arabinose-induced λ Red recombinase system that promotes recombination between linear pieces of DNA (PCR product) and the host chromosome. This recombination is based on short stretches of homology (50 nucleotides) on the linear DNA to the site of recombination. The 5' ends of the primers used for amplification of the PCR product (KO F and KO R primers) contain 50-nucleotide stretches homologous to the target gene, while the 3' ends of the primers include regions homologous to the antibiotics resistance cassette on the pKD3 or pKD4 plasmid. Each PCR product was amplified, gel extracted, and electroporated into competent strain containing pKD46 prepared with the presence of arabinose. The deletion mutants were verified by PCR using primers adjacent to the gene region (CHK F and CHK R primers) and sequencing of the PCR products.

Site-directed mutagenesis of *dnaA*

Site-directed mutagenesis of *dnaA* was performed with the corresponding primers (Table S2), using KOD-Plus-Mutagenesis Kit according to the manufacturer's recommendations (Toyobo, SMK-101). The resulting gene mutations were confirmed by DNA sequencing.

Biochemical Procedures

DnaA antibody preparation

The DnaA protein tagged with hexahistidine (6XHis) was overexpressed using pET22b-*dnaA* in *E. coli* strain BL21. The tagged protein was purified by nickel affinity chromatography under 8 M urea denaturing conditions and used to immunize rabbits for the production of antibodies.

ChIP assay

DNA fragments bound to DnaA were immunoprecipitated as described previously with some modifications [2,3]. The extracts were firstly incubated with 3% protein-A Sepharose beads for 1 h at 4°Cand then were incubated with 6 μg DnaA antibody for 4.5 h at 4°C. At last, the extracts were incubated with 3% protein-A Sepharose beads for 1 h at 4°C. The amount *oriC* in input DNA and immunoprecipitated DNA were analyzed by real-time quantitative PCR. The ratio of ChIP value to Input value (ChIP/Input) for *ylcC* was subtracted from the ChIP/Input value for *oriC* to calculate the value for specific DnaA binding. In addition, the amount of *ter* DNA product in input DNA was quantified by real-time quantitative PCR using the *ter* RT F and *ter* RT R primers, and was used to calculate the *oriC*/*ter* ratio [3].

Western blot analysis

Western blot was carried out by following standard procedures. Briefly, Proteins were resolved on 10% SDS-PAGE, transferred to PVDF membranes. For acetylation Western blot, 50 mM Tris-HCl (pH 7.5) with100 mM NaCl, 10% (V/V) Tween-20 and 1% peptone (Amresco, Solon, OH, USA) was used for blocking. 50 mM Tris-HCl (pH 7.5) with100 mM NaCl and 0.1% peptone was used to prepare primary and secondary antibodies. 50 mM Tris-HCl (pH 7.5) with 150 mM NaCl, 0.5% (V/V) Tween-20 and 5% skim milk was used for anti-DnaA and anti-His tag Western blot. Blots were scanned with G: BOX Chemi system (Syngene, Cambridge, UK), and the relative band intensities were quantified with Image J software.

Purification of YfiQ and CobB

YfiQ and CobB were overexpressed and purified by the similar procedure. BL21/pET28a- *yfiQ* and BL21/pET28a-*cobB* were induced by final concentrations of 0.1 mM IPTG at OD_{600} = 0.6

for 4 h at 37°C. Cells were harvested by centrifugation, resuspended in Binding Buffer (20 mM Tris-HCl (pH 7.6), 500 mM NaCl and 20mM imidazole), and then disrupted using an Ultrasonic Processor (Sonics, Newtown, CT, USA). The disrupted suspension was centrifuged and the resulting supernatant was loaded onto a 1-ml Ni-NTA column (GE Healthcare, USA). The column was washed initially with Washing Buffer (20 mM Tris-HCl (pH 7.6), 500 mM NaCl and 40 mM imidazole) and the histidine-tagged protein was eluted with Elution Buffer (40 mM Tris-HCl (pH 7.5), 500 mM NaCl and 500 mM imidazole). Protein purity was estimated to be > 90% on the basis of SDS-PAGE.

Purification of DnaA and its derivative mutants

For purification of DnaA, pET22b-*dnaA,* pET22b-*dnaA*(K178Q) and pET22b-*dnaA*(K178R) were constructed and confirmed by sequencing. All the constructed plasmids were introduced individually into *E. coli* strain BL21, and the resultant strains were grown in LB medium containing 100 μg/ml ampicillin at 37°C. IPTG was added to a final concentration of 0.5 mM at the time point when the absorbance of the culture at 600 nm reached 0.6-0.8. The culture was continuously incubated for 3-4 h. The cells were harvested by centrifugation and cell pellet was suspended in ice-cold PBS buffer (0.1 M Na_2HPO_4, 0.15 M NaCl, pH 7.0) containing 1.0 mM pheylmethylsulfonyl fluoride (PMSF). Cells were disrupted by using the Cell Ultrasonic system (Sonics, Newtown, CT, USA) with short bursts of 10 s followed by intervals of 30 s for cooling until they become clear. Unbroken cells and cell debris were removed by centrifugation at 21,000 x g for 40 min at 4°C. The supernatant was supplemented with 20 mM imidazole and injected into the GE Healthcare His-tag resin beans and purified by the ÄKTA™ system. His-tag resin beans were washed with 10 volume binding buffer A (20 mM sodium phosphate, 500 mM NaCl and 20 mM imidazole, pH 7.5), recombinant protein was eluted using 10 volume of buffer B (20 mM sodium phosphate, 500 mM NaCl and 500 mM imidazole, pH 7.5). The protein was checked by SDS-PAGE before shock-frozen storage at -80°C.

Purification of chromosomally encoded DnaA

BL21(His-DnaA), BW25113(His-DnaA) and their Δ*cobB*, Δ *yfiQ* and Δ*ackA* knockout derivative strains were grown to an OD_{600} of 0.2, 1.0 or overnight at 37°C in several liters of LB medium and collected. Cells were broken by high press cracker in buffer (40mM Tris-HCl, pH 7.5, 500mM NaCl, 20mM imidazole) containing DNase I. The lysate was centrifuged at 20,000 rpm

for 1 h at 4°C. The resulting supernatant was loaded onto a 1-ml Ni-NTA column (GE Healthcare, USA). The column was washed with 10 volumes of binding buffer and the bound proteins were eluted with washing buffer (40mM Tris-HCl, pH 7.5, 500 mM NaCl, 500mM imidazole as described [4]. Fractions containing DnaA were desalted using a 5ml HiTrap™ desalting column (GE Healthcare, USA).

Expression and purification of site-specific acetylated DnaA(K178Ac)

E. coli strain BL21 was transformed with plasmid pAcKRS-3 and pCDF PylT-*dnaA*(K178TAG) and then were grown overnight in LB supplemented with 50 mg/ml kanamycin and 50 mg/ml spectinomycin(LB-KS). One liter of prewarmed LB-KS was inoculated with 50 ml overnight culture and was incubated at 37°C. At OD_{600} of 1.4-1.5, the culture was supplemented with 20 mM nicotinamide (NAM) and one liter of LB with 20 mM acetyl-lysine (AcK). The final OD_{600} was 0.7-0.8 and the concentration of AcK was 10 mM. Protein expression was induced 30 min later by addition of 1 mM IPTG. Incubation was continued at 37°C, and cells were harvested 4 h after induction, washed with PBS 20 mM NAM, and stored at -80°C.

The cells were resuspended in buffer A (50 mM Tris-HCl, pH 8.0, 100 mM NaCl, 1 mM EDTA and 1% NP-40), supplemented with 20 mM NAM and DNase I and then were broken by high pressure cracker. The pellet containing inclusion body was collected by centrifugation (15 min, 18,000 rpm) and then was resuspended and washed three times in buffer B (50 mM Tris-HCl, pH 8.0, 100 mM NaCl, 2 mM EDTA and 3% TritonX-100(v/v) by ultrasonic. The pellet was macerated in buffer C (50 mM HEPES-KOH, 1 mM EDTA, 20% sucrose, 2 mM DTT, 4 M guanidinium chloride, 0.6 M ammonium sulfate, 10 mM magnesium) at room temperature for 30 min. After centrifugation (20,000 rpm, 1 h), the supernatant was gel-filtered through a Superdex 200 column equilibrated with buffer D (50 mM HEPES-KOH, 0.1 mM EDTA, 20% sucrose, 2 mM DTT, 0.2 M ammonium sulfate, 10 mM magnesium). Active fractions were pooled for using.

Protein concentration

Protein concentration was determined using the Bradford protein assay protocol (Bio-Rad, Hercules, CA, USA) with bovine serum albumin (BSA) as standard per the manufacturer's instructions.

EMSA

EMSA (Electrophoretic mobility shift assay) to test the interaction between DnaA and *oriC* was performed as described previously [5], with some modifications. The 469 bp of *oriC*-containing fragment was amplified using a pair of FAM-labeled primers, 5'-FAM-*oriC* F and 5'-FAM-*oriC* R. The indicated amounts of DnaA protein and DNA fragment were incubated for 5 min at 20°C in buffer H (20 mM HEPES-KOH, 5 mM magnesium acetate, 1 mM EDTA, 4 mM dithiothreitol, 0.2% Triton X-100, 5% (v/v) glycerol, 0.5 mg/ml BSA, and 2 mM ATP, pH 8.0). Reaction products were subjected to 5% native PAGE at 0°C. FAM-labeled fluorescence was detected by the FUJIFILM FLA7000.

Filter-binding assay

ATP- and ADP-binding activity of DnaA protein was determined by the filter-binding assay as described previously [6]. Briefly, the standard reaction(40μl) containing buffer G (Tricine-KOH (pH 8.25 at 1 M), 50 mM; magnesium acetate, 2.5 mM; EDTA, 0.3 mM; glycerol, 20% (v/v); Triton X-100, 0.007%; dithiothreitol, 7 mM); [α-^{32}P]ATP or [^{3}H]ADP; DnaA (130ng) was incubated at 0°C for 15 min. Samples were filtered through 0.45 μm nitrocellulose membranes (Millipore HA, USA) presoaked in wash buffer (50 mM Tricine-KOH (pH 8.25), 0.5 mM magnesium acetate, 0.3 mM EDTA, 5 mM dithiothreitol, 17% (v/v) glycerol, 10 mM ammonium sulfate, and 0.005% Triton X-100). Every filter was washed with 6 ml of ice-cold wash buffer three times and dried. The radioactivity remaining on the filters was counted in a liquid scintillation counter.

Identification of acetylated lysine residues by mass spectrometry

The purified chromosomally encoded DnaA was separated by 10% one-dimensional SDS-PAGE and the bands containing DnaA were excised. The excised bands were destained and dehydrated. For trypsin digestion, proteins were treated with 100 mM DTT at 56°C for 30 min and then treated with 100 mM NH_4HCO_3 at room temperature for 15 min. The freeze-dried samples were incubated with 100-200 ng trypsin at 37°C for 20 h. Peptides generated after proteolytic digestion of DnaA were separated by the EASY-nLC HPLC system (Thermo Scientific, USA) and analyzed by Q-Exactive mass spectrometer (Thermo Scientific, USA). Mass spectrometric data were analyzed using the Mascot 2.2 software for database search.

Real-Time quantitative PCR

Cells at the early logarithmic (EL) phase, late logarithmic (LL) phase and stationary phase were harvested by centrifuging at 8,000 x g for 10 min at 4°C. Extracts of DNA (2 μg) were analyzed by ABI PRISM 7500 fast Sequence Detection System (Applied Biosystems). The primers include: *oriC* RT (F-; R-), *ter* RT (F-; R-) and 16S rDNA RT (F-; R-) (Table S2). The PCR reactions were carried out under the following conditions: 95°C for 1 minute, 40 cycles at 95°C for 15 s, 60°C for 15 s, and 72°C for 30 s. SyberGreen real-time quantitative PCR dissociation curves showed that each primer set gave a single and specific product. The 16S rDNA was used as internal standards to adjust for different qualities and quantities of DNA. All data are expressed as mean ± SEM.

Supplementary Tables

Supplementary Table S1 Strains and plasmids used in this study

**Strains or plasmids**	**Description**	**Sources**
**Strains**		
E. coli DH5α	F- 80*lacZ* M15 (lacZYA–argF)U169 *eoR* *recA1endA1 hsdR17 phoA supE*44-thi-1 *gyrA96 relA1*	Laboratory stock
E. coli BL21	F- *ompT gal dcm lon hsdSB* (rB- mB-) λ(DE3 [*lacI lacUV*5-T7 gene 1 *ind1 sam7 nin5*])	Laboratory stock
BL21 derivatives		
Δ*cobB*	[BL21]Δ*cobB*	This work
Δ*yfiQ*	[BL21]Δ*yfiQ*	This work
Δ*ackA*	[BL21]Δ*ackA*	This work
his-dnaA	[BL21]*his-dnaA*(cm^R)	This work
Δ*cobB-his-dnaA*	[BL21]Δ*cobB-his-dnaA*(cm^R)	This work
Δ*yfiQ-his-dnaA*	[BL21]Δ*yfiQ-his-dnaA*(cm^R)	This work
Δ*ackA-his-dnaA*	[BL21]Δ*ackA-his-dnaA*(cm^R)	This work
E. coli BW25113	$LacI^q$ $rrnB_{T14}$ $\Delta lacZ_{WJ16}$*hsdR514* $\Delta(araBAD)_{AH33}$ $\Delta(rhaBAD)_{LD78}$	Laboratory stock
BW25113 derivatives		

Δ*cobB*	[BW25113] Δ*cobB::kan*	Laboratory stock
Δ*yfiQ*	[BW25113] Δ*yfiQ::kan*	Laboratory stock
Δ*ackA*	[BW25113] Δ*ackA::kan*	Laboratory stock
his-dnaA	[BW25113] *his-dnaA*(cm^R)	
Δ*cobB-his-dnaA*	[BW25113] *cobB::kan-his-dnaA*(cm^R)	This work
Δ*yfiQ-his-dnaA*	[BW25113] Δ*yfiQ::kan-his-dnaA*(cm^R)	This work
Δ*ackA-his-dnaA*	[BW25113]Δ*ackA::kan-his-dnaA*(cm^R)	This work
E. coli MG1655	*F- λ- ilvG- rfb-50 rph-1*	Laboratory stock
E. coli KA413	*Ilv thyA tyrA*(Am) *trpE9829*(Am) *metE deo supF6*(Ts) *dnaA46*	Gift from T. Katayama
**Plasmids**		
pCDSSara	Spe^R , used for plasmid complementation assay	Gift from Peter Schultz
pCDSSara-*dnaA*	pCDSSara harboring wild type *dnaA*	This work
pCDSSara-*dnaA*(K81Q)	pCDSSara harboring *dnaA*(K81Q)	This work
pCDSSara-*dnaA*(K81R)	pCDSSara harboring *dnaA*(K81R)	This work
pCDSSara-*dnaA*(K135Q)	pCDSSara harboring *dnaA*(K135Q)	This work
pCDSSara-*dnaA*(K135R)	pCDSSara harboring *dnaA*(K135R)	This work
pCDSSara-*dnaA*(K145Q)	pCDSSara harboring *dnaA*(K145Q)	This work
pCDSSara-*dnaA*(K145R)	pCDSSara harboring *dnaA*(K145R)	This work
pCDSSara-*dnaA*(K178Q)	pCDSSara harboring *dnaA*(K178Q)	This work

pCDSSara-*dnaA*(K178R)	pCDSSara harboring *dnaA*(K178R)	This work
pCDSSara-*dnaA*(K197Q)	pCDSSara harboring *dnaA*(K197Q)	This work
pCDSSara-*dnaA*(K197R)	pCDSSara harboring *dnaA*(K197R)	This work
pCDSSara-*dnaA*(K212Q)	pCDSSara harboring *dnaA*(K212Q)	This work
pCDSSara-*dnaA*(K212R)	pCDSSara harboring *dnaA*(K212R)	This work
pCDSSara-*dnaA*(K223Q)	pCDSSara harboring *dnaA*(K223Q)	This work
pCDSSara-*dnaA*(K223R)	pCDSSara harboring *dnaA*(K223R)	This work
pCDSSara-*dnaA*(K308Q)	pCDSSara harboring *dnaA*(K308Q)	This work
pCDSSara-*dnaA*(K308R)	pCDSSara harboring *dnaA*(K308R)	This work
pCDSSara-*dnaA*(K309Q)	pCDSSara harboring *dnaA*(K309Q)	This work
pCDSSara-*dnaA*(K309R)	pCDSSara harboring *dnaA*(K309R)	This work
pCDSSara-*dnaA*(K327Q)	pCDSSara harboring *dnaA*(K327Q)	This work
pCDSSara-*dnaA*(K327R)	pCDSSara harboring *dnaA*(K327R)	This work
pCDSSara-*dnaA*(K381Q)	pCDSSara harboring *dnaA*(K381Q)	This work
pCDSSara-*dnaA*(K381R)	pCDSSara harboring *dnaA*(K381R)	This work
pCDSSara-*dnaA*(K390Q)	pCDSSara harboring *dnaA*(K390Q)	This work
pCDSSara-*dnaA*(K390R)	pCDSSara harboring *dnaA*(K390R)	This work
pCDSSara-*dnaA*(K397Q)	pCDSSara harboring *dnaA*(K397Q)	This work
pCDSSara-*dnaA*(K397R)	pCDSSara harboring *dnaA*(K397R)	This work
pCDSSara-*dnaA*(K443Q)	pCDSSara harboring *dnaA*(K443Q)	This work
pCDSSara-*dnaA*(K443R)	pCDSSara harboring *dnaA*(K443R)	This work

pCDSSara-*dnaA*(K455Q)	pCDSSara harboring *dnaA*(K455Q)	This work
pCDSSara-*dnaA*(K455R)	pCDSSara harboring *dnaA*(K455R)	This work
pET22b-*dnaA*	Amp^R, used for DnaA expression	This work
pET22b-*dnaA*(K178Q)	Amp^R, used for DnaA(K178Q) expression	This work
pET22b-*dnaA*(K178R)	Amp^R, used for DnaA(K178R) expression	This work
pAcKRS-3	Kan^R, coding for *M. barkeri* pyrrolysine tRNA synthetase	Gift from J Chin
pCDF-PylT	Spe^R, derivated from pCDFduet and harboring the *pylT* gene	Gift from J Chin
pCDF-PylT-*dnaA*	Spe^R, used for protein expression	This work
pCDF-PylT-*dnaA* (K178TAG)	Spe^R, used for acetylated DnaA expression	This work
pBKS-*oriC*	Amp^R, derivated from pBluescript II KS(+) and containing the 467bp-*oriC* fragment	This work
pGEX4T-1	Amp^R, used for pull-down assay	This work
pGEX4T-1-*cobB*	Amp^R, used for pull-down assay	This work
pGEX4T-1-*yfiQ*	Amp^R, used for pull-down assay	This work

Supplementary Table S2 Primers used in this study

Primer Name	Primer Sequence
cobB-KO F	TGCGTGGTGCGGCCTTCCTACATCTAACCGATTAAACAACAGAG GTTGCTGTGTAGGCTGGAGCTGCTTC
cobB-KO R	CGCAAATTCAATTAATTGCGTCCCCTTGCAGGCCTGATAAGCGT AGTGCACATATGAATATCCTCCTTA
cobB CHK F	CTCGTCATCTCTTACCTGTA
cobB CHK R	AGCTTTGCGATTACCAGAATA
yfiQ-KO F	ATGAGTCAGCGAGGACTGGAAGCACTACTGCGACCAAAATCGA TAGCGGTGTGTAGGCTGGAGCTGCTTC
yfiQ-KO R	TGGGCAAGATTTAGCGTAAGCCCAACGATCCCCTCTTCGAGCT GGATATCCATATGAATATCCTCCTTA
yfiQ CHK F	AGGCTGAAGGCCAATATTGT
yfiQ CHK R	GGGAGCGTAAAAGAAATCGA
ackA-KO F	CTATGGCTCCCTGACGTTTTTTTAGCCACGTATCAATTATAGGTA CTTCCGTGTAGGCTGGAGCTGCTTC
ackA-KO R	GATTTGGCGGGTTACAAAACAGCACCGCCAGCTGAGCTGGCG GTGTGAAACATATGAATATCCTCCTTA
ackA CHK F	AATGGCATAGACTCAAGATA
ackA CHK R	GGTTTATCCTCTTTCGTTAC
His-DnaA F1	TTACTTAGAAACGGTCAGAC

His-DnaA R1	GAAGCAGCTCCAGCCTACACTAGACAAAAATTGGCTTAAT
His-DnaA F2	ATTAAGCCAATTTTTGTCTAGTGTAGGCTGGAGCTGCTTC
His-DnaA R2	ATTGGAAAATTTAATGACCACCATATGAATATCCTCCTTA
His-DnaA F3	TAAGGAGGATATTCATATGGTGGTCATTAAATTTTCCAAT
His-DnaA R3	ATGATGATGATGATGATGCACGGCGGACTCCACTCGAACAA
His-DnaA F4	TTGTTCGAGTGGAGTCCGCCGTGCATCATCATCATCATCATTCA CTTTCGCTTTGGCAGCA
pCDSSara-*dnaA* F	CCGCTCGAGGTGTCACTTTCGCTTTGGC
pCDSSara-*dnaA* R	GGACTAGTTTACGATGACAATGTTCTG
K81Q F	TTTGAAGTCGGCACCCAACCGGTGACGCAAACG
K81Q R	CGTTTGCGTCACCGGTTGGGTGCCGACTTCAAA
K81R F	TTTGAAGTCGGCACCCGACCGGTGACGCAAACG
K81R R	CGTTTGCGTCACCGGTCGGGTGCCGACTTCAAA
K135Q F	TCTAACGTAAACGTCCAACACACGTTTGATAAC
K135Q R	GTTATCAAACGTGTGTTGGACGTTTACGTTAGA
K135R F	TCTAACGTAAACGTCCGACACACGTTTGATAAC
K135R R	GTTATCAAACGTGTGTCGGACGTTTACGTTAGA
K145Q F	AACTTCGTTGAAGGTCAATCTAACCAACTGGCG
K145Q R	CGCCAGTTGGTTAGATTGACCTTCAACGAAGTT
K145R F	AACTTCGTTGAAGGTCGATCTAACCAACTGGCG
K145R R	CGCCAGTTGGTTAGATCGACCTTCAACGAAGTT

K178Q F	GGCACGGGTCTGGGTCAAACTCACCTGCTGCAT
K178Q R	ATGCAGCAGGTGAGTTTGACCCAGACCCGTGCC
K178R F	GGCACGGGTCTGGGTCGAACTCACCTGCTGCAT
K178R R	ATGCAGCAGGTGAGTTCGACCCAGACCCGTGCC
K197Q F	CGCAAGCCGAATGCCCAAGTGGTTTATATGCAC
K197Q R	GTGCATATAAACCACTTGGGCATTCGGCTTGCG
K197R F	CGCAAGCCGAATGCCCGAGTGGTTTATATGCAC
K197R R	GTGCATATAAACCACTCGGGCATTCGGCTTGCG
K212Q F	GTTCAGGACATGGTTCAAGCCCTGCAAAACAAC
K212Q R	GTTGTTTTGCAGGGCTTGAACCATGTCCTGAAC
K212R F	GTTCAGGACATGGTTCGAGCCCTGCAAAACAAC
K212R R	GTTGTTTTGCAGGGCTCGAACCATGTCCTGAAC
K223Q F	CGATCGAAGAGTTTCAACGCTACTACCGTTCC
K223Q R	GGAACGGTAGTAGCGTTGAAACTCTTCGATCG
K223R F	CGATCGAAGAGTTTCGACGCTACTACCGTTCC
K223R R	GGAACGGTAGTAGCGTCGAAACTCTTCGATCG
K308Q F	GTGGCGATCCTGATGCAAAAGGCCGACGAAAAC
K308Q R	GTTTTCGTCGGCCTTTTGCATCAGGATCGCCAC
K308R F	GTGGCGATCCTGATGCGAAAGGCCGACGAAAAC
K308R R	GTTTTCGTCGGCCTTTCGCATCAGGATCGCCAC
K309Q F	GCGATCCTGATGAAACAGGCCGACGAAAACGAC

K309Q R	GTCGTTTTCGTCGGCCTGTTTCATCAGGATCGC
K309R F	GCGATCCTGATGAAACGGGCCGACGAAAACGAC
K309R R	GTCGTTTTCGTCGGCCCGTTTCATCAGGATCGC
K327Q F	GCGTTCTTTATCGCCCAGCGTCTACGATCTAAC
K327Q R	GTTAGATCGTAGACGCTGGGCGATAAAGAACGC
K327R F	GCGTTCTTTATCGCCCGGCGTCTACGATCTAAC
K327R R	GTTAGATCGTAGACGCCGGGCGATAAAGAACGC
K381Q F	ATCGACAATATTCAGCAGACGGTGGCGGAGTAC
K381Q R	GTACTCCGCCACCGTCTGCTGAATATTGTCGAT
K381R F	ATCGACAATATTCAGCGGACGGTGGCGGAGTAC
K381R R	GTACTCCGCCACCGTCCGCTGAATATTGTCGAT
K390Q F	GAGTACTACAAGATCCAAGTCGCGGATCTCCTT
K390Q R	AAGGAGATCCGCGACTTGGATCTTGTAGTACTC
K390R F	GAGTACTACAAGATCCGAGTCGCGGATCTCCTT
K390R R	AAGGAGATCCGCGACTCGGATCTTGTAGTACTC
K397Q F	GCGGATCTCCTTTCCCAGCGTCGATCCCGCTCG
K397Q R	CGAGCGGGATCGACGCTGGGAAAGGAGATCCGC
K397R F	GCGGATCTCCTTTCCCGGCGTCGATCCCGCTCG
K397R R	CGAGCGGGATCGACGCCGGGAAAGGAGATCCGC
K443Q F	GCTTCATGCCTGCCGTAAGATCGAGCAGTTGCGTG
K443Q R	CACGCAACTGCTCGATCTTACGGCAGGCATGAAGC

K443R F	GCTTCATGCCTGCCGTCGGATCGAGCAGTTGCGTG
K443R R	CACGCAACTGCTCGATCCGACGGCAGGCATGAAGC
K455Q F	GAGAGCCACGATATCCAAGAAGATTTTTCAAAT
K455Q R	ATTTGAAAAATCTTCTTGGATATCGTGGCTCTC
K455R F	GAGAGCCACGATATCCGAGAAGATTTTTCAAAT
K455R R	ATTTGAAAAATCTTCTCGGATATCGTGGCTCTC
K178Ac F	GGCACGGGTCTGGGTTAGACTCACCTGCTGCAT
K178Ac R	ATGCAGCAGGTGAGTCTAACCCAGACCCGTGCC
pET22b-*dnaA* F	TCTCCATTAGGTGTCACTTTCGCTTTGG
pET22b-*dnaA* R	TCTCCTCGAGCGATGACAATGTTCTGAT
pBKS-*oriC* F	CCGGAGCTCGCCAATGATGATGACGTCAA
pBKS-*oriC* R	GGGGTACCAATCGGTATTGGTAGTCGTG
pCDF-*dnaA* F	CCGCCATGGGCCATCATCATCATCATCATTCACTTTCGCTTTGG CAGCA
pCDF-*dnaA* R	GGCTCGAGTTACGATGACAATGTTCTGATT
5'FAM-*oriC* F	5'FAM-CCCGGGCCGTGGATTCTACT
5'FAM-*oriC* R	5'FAM-CTCGAGGCAGAACTCAAAGA
oriC RT F	CTGTGAATGATCGGTGATC
oriC RT R	GTGGATAACTCTGTCAGGAAGCTTG
ter RT F	TCCTCGCTGTTTGTCATCTT
ter RT R	GGTCTTGCTCGAATCCCTT

ylcC RT F	GGCGTGGTAAAGGGTATCG
ylcC RT R	TCTGCGGGGTGATGGTAAAG
16S rDNA RT F	CAGCCACACTGGAACTGAGA
16S rDNA RT R	GTGCTTCTTCTGCGGGTAAC

Supplementary Table S3 The plasmid complementation assay

Plasmid	Allele	Transformation efficiency (CFU/µg DNA)		
		30°C	42°C	42°C/30°C
pCDSSara-*dnaA*	WT	$4.80*10^6$	$5.60*10^6$	1.17
pCDSSara-*dnaA*(K81Q)	K81Q	$9.20*10^5$	$1.44*10^6$	0.64
pCDSSara-*dnaA*(K81R)	K81R	$2.52*10^5$	$3.16*10^5$	0.80
pCDSSara-*dnaA*(K135Q)	K135Q	$4.20*10^5$	$5.20*10^5$	1.24
pCDSSara-*dnaA*(K135R)	K135R	$2.64*10^5$	$4.72*10^5$	1.79
pCDSSara-*dnaA*(K145Q)	K145Q	$4.00*10^7$	$4.00*10^7$	1.00
pCDSSara-*dnaA*(K145R)	K145R	$1.13*10^9$	$1.22*10^9$	1.08
pCDSSara-*dnaA*(K178Q)	K178Q	$9.70*10^7$	0	0
pCDSSara-*dnaA*(K178R)	K178R	$4.86*10^6$	0	0
pCDSSara-*dnaA*(K197Q)	K197Q	$0.72*10^5$	$0.56*10^5$	0.73
pCDSSara-*dnaA*(K197R)	K197R	$2.20*10^5$	$2.64*10^5$	1.20
pCDSSara-*dnaA*(K212Q)	K212Q	$1.03*10^9$	$0.91*10^9$	0.88
pCDSSara-*dnaA*(K212R)	K212R	$4.00*10^8$	$4.8*10^8$	1.20
pCDSSara-*dnaA*(K223Q)	K223Q	$2.48*10^6$	$3.36*10^6$	1.35
pCDSSara-*dnaA*(K223R)	K223R	$8.72*10^6$	$9.72*10^6$	1.11
pCDSSara-*dnaA*(K308Q)	K308Q	$5.68*10^5$	$6.79*10^5$	1.19
pCDSSara-*dnaA*(K308R)	K308R	$1.08*10^5$	$1.72*10^5$	1.59

pCDSSara-*dnaA*(K309Q)	K309Q	$4.20*10^{5}$	$2.88*10^{5}$	0.68
pCDSSara-*dnaA*(K309R)	K309R	$3.68*10^{5}$	$2.68*10^{5}$	0.73
pCDSSara-*dnaA*(K327Q)	K327Q	$2.20*10^{6}$	$3.08*10^{6}$	0.71
pCDSSara-*dnaA*(K327R)	K327R	$8.80*10^{5}$	$9.20*10^{5}$	0.96
pCDSSara-*dnaA*(K381Q)	K381Q	$5.44*10^{6}$	$9.09*10^{6}$	0.60
pCDSSara-*dnaA*(K381R)	K381R	$1.78*10^{7}$	$4.56*10^{6}$	0.39
pCDSSara-*dnaA*(K390Q)	K390Q	$2.4*10^{6}$	$4.7*10^{6}$	1.96
pCDSSara-*dnaA*(K390R)	K390R	$7.2*10^{6}$	$5.4*10^{6}$	0.75
pCDSSara-*dnaA*(K397Q)	K397Q	$3.36*10^{6}$	$3.04*10^{6}$	1.11
pCDSSara-*dnaA*(K397R)	K397R	$1.04*10^{6}$	$3.12*10^{6}$	0.33
pCDSSara-*dnaA*(K443Q)	K443Q	$5.52*10^{5}$	$4.40*10^{5}$	1.25
pCDSSara-*dnaA*(K443R)	K443R	$5.12*10^{6}$	$5.12*10^{6}$	1.00
pCDSSara-*dnaA*(K455Q)	K455Q	$6.68*10^{6}$	$6.84*10^{6}$	1.02
pCDSSara-*dnaA*(K455R)	K455R	$3.60*10^{6}$	$3.60*10^{6}$	1.00
pCDSSara-vector	None	$6.40*10^{6}$	0	0

KA413 (*dnaA*46[Ts]) cells bearing the indicated plasmid were grown overnight at 30°C in LB agar containing thymine (50 μg/mL) and spe (100 μg/mL), at 42°C on LB agar containing thymine (50 μg/mL) and spe (100 μg/mL) and 10 mM Ara.

Supplementary Figures

Supplementary Figure S1

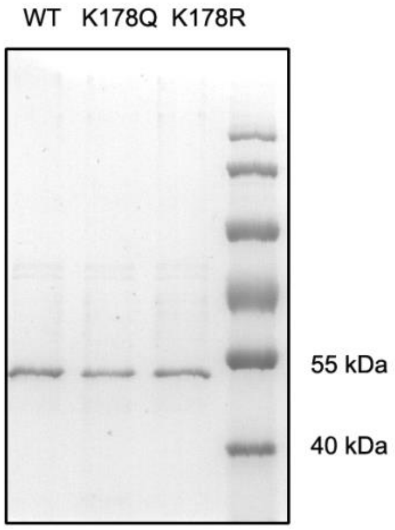

Purification of DnaA, DnaA K178Q and DnaA K178R. The overexpressed DnaA and its mutants were purified and desalted as described in “Materials and Methods”. K178Q, K178R and the wild-type DnaA protein (250 ng) were resolved on 10% SDS-PAGE and stained with Coomassie blue.

Supplementary Figure S2

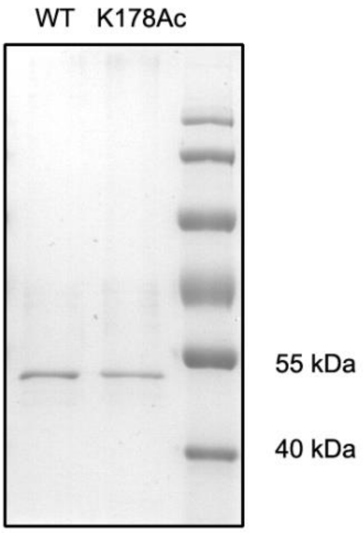

The purification of DnaA K178Ac. DnaA and DnaA K178Ac were expressed and purified as described in “Materials and Methods”. K178Ac and the wild-type DnaA protein (200 ng) were resolved on 10% SDS-PAGE and stained with Coomassie blue.

Supplementary Figure S3

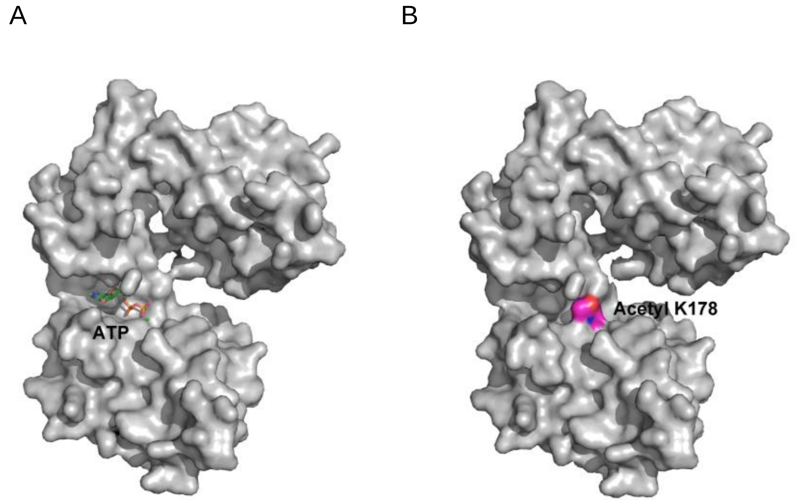

Modeling of structure of the *E. coli* DnaA. The homology structure of the *E. coli* DnaA was modeled by Modeller 9.11, using the crystal structure of DnaA of *Aquifex aeolicus* (1L8Q) from RSC Protein Data Bank as its template. The acetyl K178 was modified and further optimized by Sybyl 6.8. Panel A showed ATP bound in the substrate pocket and the surrounding residues of DnaA. Panel B showed the acetylated K178 had a steric clash with the phosphate group of ATP.

Supplementary Figure S4

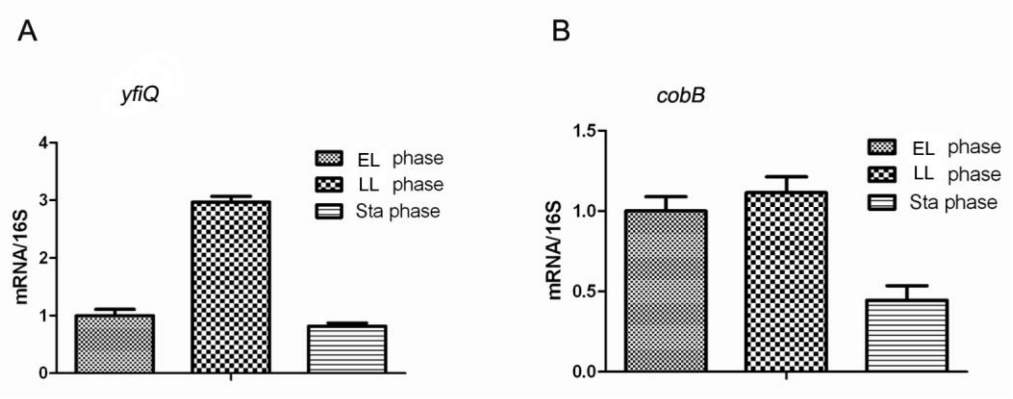

Quantification of mRNA levels of *yfiQ* and *cobB* in *E. coli* at different growth phases. (A) Relative *yfiQ* mRNA levels at different growth phases. (B) Relative *cobB* mRNA levels at different growth phases. Total RNA was isolated from *E. coli* strain BW25113 grown at early logarithmic (EL), late logarithmic (LL) or stationary phase in LB media at 37°C and used for the real-time qPCR analysis. The 16S rDNA was used as internal standard.

Supplementary Methods References:

1 Datsenko, K. A. & Wanner, B. L. One-step inactivation of chromosomal genes in Escherichia coli K-12 using PCR products. *Proceedings of the National Academy of Sciences of the United States of America* **97**, 6640-6645, doi:10.1073/pnas.120163297 (2000).

2 Lin, D. C. & Grossman, A. D. Identification and characterization of a bacterial chromosome partitioning site. *Cell* **92**, 675-685 (1998).

3 Kasho, K. & Katayama, T. DnaA binding locus datA promotes DnaA-ATP hydrolysis to enable cell cycle-coordinated replication initiation. *Proceedings of the National Academy of Sciences of the United States of America* **110**, 936-941, doi:10.1073/pnas.1212070110 (2013).

4 Liang, W., Malhotra, A. & Deutscher, M. P. Acetylation regulates the stability of a bacterial protein: growth stage-dependent modification of RNase R. *Molecular cell* **44**, 160-166, doi:10.1016/j.molcel.2011.06.037 (2011).

5 Kawakami, H., Keyamura, K. & Katayama, T. Formation of an ATP-DnaA-specific initiation complex requires DnaA Arginine 285, a conserved motif in the AAA+ protein family. *The Journal of biological chemistry* **280**, 27420-27430, doi:10.1074/jbc.M502764200 (2005).

6 Nishida, S. *et al.* A nucleotide switch in the Escherichia coli DnaA protein initiates chromosomal replication: evidnece from a mutant DnaA protein defective in regulatory ATP hydrolysis in vitro and in vivo. *The Journal of biological chemistry* **277**, 14986-14995, doi:10.1074/jbc.M108303200 (2002).
